# Editorial, Notes and Miscellany

**Published:** 1911-05

**Authors:** 


### Editorial, Notes and Miscellany.

Dr. Geo. Harwood has removed from Sabinal to Johnson City, Texas.

Dr. T. J. Largen, of San Antonio—one of the old guard—died April 24, aged 70 years.

Married at Dallas, Texas, April 19 (ult.), Dr. Edward H. Carey to Miss Georgie Fonda Schneider, all of Dallas. Congratulations!

Another Benedict.—Dr. H. K. Beall, of Fort Worth, member of the Texas State Board of Health, was married April 19 to Miss Camilla Labatt, of that city.

The American Society of Tropical Medicine.—The eighth annual meeting of the American Society of Tropical Medicine will be held at the Tulane University, New Orleans, La., U. S. A., May 18 and 19.

A Valuable Premium.—Any person who will send me three new subscribers and $3.00 will receive Dr. Bruno by mail, free, or if a subscriber his paid subscription will be extended three years. Everybody is curious to see this wonderful book; selling like hot cakes.

The Texas State Medical Association will meet at Amarillo, Texas, May 9, 10 and 11. As the program appeared in the Association journal for April, and as all my Texas subscribers get the journal, whether they want it or not, I only mention the date of meeting.

The forty-second annual meeting of the American Medical Editors' Association will be held at Los Angeles, Cal., June 26 and 27 (prox.). Dr. Joseph MacDonald, Jr., of the *American Journal of Surgery,* New York, is President, and Dr. J. J. Taylor, of the *Medical World,* Philadelphia, is Secretary and Treasurer.

"Let us therefore boldly face the life of strife, resolute to do our duty well and manfully; resolute to uphold righteousness by deed and word; resolute to be both honest and brave, to serve high ideals, yet to use practical methods. Above all, let us shrink from no strife, moral or physical, within or without the nation, provided we are certain that the strife is justified, for it is only through strife, through hard and dangerous endeavor, that we shall ultimately win the goal of true national happiness."—Theodore Roosevelt.

---

A board of commissioned medical officers will be convened to meet at the Bureau of Public Health and Marine Hospital Service, 3 B Street, SE., Washington, D. C., May 22, 1911, at 10 o'clock a. m., for the purpose of examining candidates for admission to the grade of assistant surgeon in the Public Health and Marine Hospital Service.

Candidates must be between 22 and 30 years of age, graduates of a reputable medical college, and must furnish testimonials from responsible persons as to their professional and moral character.

---

There is said to be an aura, or atmosphere, surrounding each one of us, and the electrical negative or positive force emanating from this makes us magnetic or antagonistic to our fellowmen. Perhaps it is more a question of an understanding mind than an atmosphere, and a sympathetic soul than an electrical current. There are latent forces in the human soul, which, when known and understood, can be developed and used to control the worst in us and exalt the best. First we creep, then we crawl, then we stand alone, and finally we walk. This is typical of mental and moral, as well as physical development.—Jos. Collins, in *Medical Record*.

---

For Sale or Exchange for city property, 1700 acres in famous San Dequito American colony, 100 miles west of Tampico. Railroad station on property; permanent water, no irrigation necessary; 100 acres or more cleared; two acres in winter tomatoes, now shipping to the United States. Neighbors sold sugar cane in field uncut for $50 gold per acre. Sugar mills nearby. Some thirty families of natives living on the property—renters. Most beautiful location for town between San Luis Potosi and Tampico. About 700 acres valley lying along the railroad; balance hill and mountain. Will sell valley alone if preferred, or whole tract; basis, $10 gold. Address, Editor TEXAS MEDICAL JOURNAL, Austin, Texas.

---

ASSOCIATION OF MEDICAL OFFICERS OF THE ARMY AND NAVY OF THE CONFEDERACY.—The twenty-first annual reunion of the United Confederate Veterans will be held at Little Rock, Ark., May 16, 17 and 18, 1911.

At the same time and place will also convene the fourteenth annual meeting of the Association of Medical Officers of the Army and Navy of the Confederacy. Dr. Edwin D. Newton, of Atlanta, Ga., will preside. Dr. Newton enjoys the unique distinction of being the only surviving officer attached to the medical staff of General R. E. Lee's headquarters.

---

THE STATE BOARD OF HEALTH, under the leadership of President Ralph Steiner, has just consummated arrangements for the distribution of diphtheria antitoxin. It is superfluous to indicate to any member of the medical profession the immunizing and curative value of diphtheria antitoxin. That plans for its general distribution, at exceedingly low prices, have been perfected is of the greatest moment.

The arrangements just completed provide that the State Health Board shall have direct supervision of the distribution of the antitoxin, but shall not handle the receipts for same. It is desired that city councils and county commissioners see to the distribution of antitoxin among the poor. The plan at once to be placed in operation includes the making of clinical reports of all cases treated, whether immunizing or curative.—*Bulletin State Board of Health.*

---

THE TEXAS LEPROSARIUM is to be established. Two years ago the Legislature passed a bill for such an institution and appropriated $40,000 for the purpose. It became a law without the Governor's signature, but he did nothing to put it into effect. Governor Colquitt has revived it and appointed Dr. M. M. Carrick, of Dallas, late Assistant Superintendent of the Epileptic Colony at Abilene, to be Superintendent,—an excellent and wise selection. He has also appointed W. E. Gilliland, of Baird, Rev. R. J. Briggs, of Austin, and Dr. Ralph Steiner, President State Board of Health, ex-officio, to be the commissioners to locate the Leper Home. It must be five miles from any town and one mile from any house or home. Dr. Carrick is a handsome bachelor, and it looks cruel to hide him away out there where the girls have no chance to catch him. Serves him right for being a bachelor.

---

IMPORTANCE OF FUN.—Nobody runs from fun. As an exchange said recently:

"A world without humor would be a fleeting show for man's delusion given.

"Humor is one of the greatest compensations of life. We may be able to do everything else by rule, but we shall never learn to laugh by rule. Philosophy is deceitful, psychology is vain, but humor is true and certain.

"Humor makes motions, introduces bills, addresses assemblies and brings important business before the house. It is cosmopolitan and a home missionary. When a heart is heavy as a Saratoga trunk, the first smile is the presence of the relief corps. At the first suggestion of suffering humor is instantly transformed into sympathy and, with red cross on its arm, rushes to the rescue.

"Humor may be enslaved and degraded, but it only shares the fate of any high spirit which can be confiscated and made to serve the lowest ends. Newspapers are the first aid to the injured by being the purveyors of humor, but impure humor has no standing among American newspapers."—*Ex.*

---

A REAL RIP VAN WINKLE.—THE STRANGE CASE OF DR. BRUNO. By F. E. Daniel, M. D., Sold by Von Boeckmann-Jones Company, Austin, Texas. Price, $1.50, or $2.00 with the "Red Back" one year.

Dr. Daniel, the well known editor of the Texas "Red Back" (the *Texas Medical Journal*), has had a quarter of a century of experience as a medical writer, and now, with amazing versatility, he gives us an intensely interesting work of fiction teeming with heart interest. He has blended with the hand of a master, philosophy, religious faith, scientific knowledge, love, romance, and cruelty into an imaginative tale which enthralls the reader from the first page to the close. We were annoyed when compelled to lay it down during our reading, and impatient till we got back to it. The possibility of a synthetic drug inducing prolonged suspended animation is the central theme around which he weaves a weird romance. Well known scientific facts are so deftly threaded among physical impossibilities that the reader must be on his guard lest he dream that astounding discoveries are actually being exemplified and analyzed as demonstrable truths. One feels that the mantle of Jules Verne or Rider Haggard has fallen on worthy shoulders. The doctor who starts to read this book will neglect some of his patients. We know it has thrilled us in a way we have never been thrilled since we first read Ben Hur. The owner will always have a book that he may hand to a friend in assurance that it will be thoroughly enjoyed; but if it is left on the waiting-room table it will surely be stolen.—A. L. R., in *Medical World,* Philadelphia.

---

THE REFORMER.

(With apologies to Benton Bradley—see February *World,* page 78.)

Yes, it made me *mad* when I read about
That "poor, starved devil who flickered out,"
And I pointed out why he had no chance
"In the tangled meshes of circumstance."
But he turned on me a contemptuous grin
And he told me to go and wipe off my chin.

Oh! It made me *rage* when I came to learn
"Of a clean-souled woman who could not earn
Enuf to live"; and I tried to tell
Why the fight was fierce and why she fell.
But it made her "*seethe* with an anger hot,"
And she broke my head with an old crackt pot.

In the holy place I saw the Beast,
Protected by dupes in his wicked feast,
They "stript the tree of its goodly fruit."
'Twas his victims that pampered the ravening brute.
They armed the tryant with all his might—
For him they preyed by day and by night.

My *wrath* flamed hot "at the sight of pain,
Of needless sorrow and heedless gain,"
So I tried to enter a mild protest
On behalf of the ravisht and opprest;
But those who knew poverty, shame, and gall—
The wounded and bleeding ones doomed to fall—
With hideous outcry they charged at me;
Like Sancho Panzy, I climbed a tree.

—J. A. Mulnix, M. D., in *Medical World.*

### THE WONDERFUL HAIRPIN.

Whenever her switch would grow suddenly loose
  She would fasten it up with a hairpin;
And if her belt buckle grew too weak for use,
  She would fasten it up with a hairpin.
Of mornings, when she wished to open her mail,
Or if in a magazine she read a tale
And wished to cut pages, this maid young and frail
  Reached up in her hair for a hairpin.

A man might call for a whole box of tools—
  She simply reached up for a hairpin;
A man might spend years in mechanical schools
  To learn what she did with a hairpin;
A man would get flustered, and frown, and perspire,
And ask who the dickens had taken his wire
When for some repairing such stuff he'd require—
  She always relied on a hairpin.

A scissors, a knife, or a tweezers or awl—
  She did very well with a hairpin.
She found that the stairway that rose from the hall
  Was measured quite well with a hairpin;
An egg beater broken? A laundry pipe plugged?
A corkscrew not found? Then her shoulders she shrugged
And reached, while her sense of contentment she hugged,
  Right up to her hair for a hairpin.

A manicure set, and a button hook, too,
  She always could find in a hairpin;
In fact, there was nothing a person could do
  That she couldn't do with a hairpin.
One day she was wrecked in a passenger train;
The crew cried: "We'll have to send back for a crane!"
She murmured, her arm through a cracked window pane:
  "Don't bother. I'll lend you a hairpin."

—*Ex.*

---

When resecting the cecum be careful not to tie the mesentery of the ascending colon too close to the bowel—the anastomosing loop of the ileo-colic and colica media lies very near the gut.—*American Journal of Surgery.*

